# Nursing workload and severity of COVID-19 patients in the Intensive Care Unit

**DOI:** 10.1590/1980-220X-REEUSP-2024-0107en

**Published:** 2024-08-23

**Authors:** Wesley Cajaiba Santos, Maria Carolina Barbosa Teixeira Lopes, Cassia Regina Vancini-Campanharo, Daniela Boschetti, Sirlei Oliveira da Silva Dias, Meire Cristina Novelli e Castro, Luis Humberto Vieri Piacezzi, Ruth Ester Assayag Batista

**Affiliations:** 1 Universidade Federal de São Paulo, Escola Paulista de Enfermagem, São Paulo, SP, Brazil.; 2 Hospital e Maternidade do SEPACO, Serviço Social da Indústria do Papel, Papelão e Cortiça do Estado de São Paulo, São Paulo, SP, Brazil.; 3 Faculdade de Medicina da Universidade Estadual Paulista Júlio de Mesquita Filho, Departamento de Enfermagem, Botucatu, SP, Brazil.

**Keywords:** Nurses, Workload, Patient Acuity, Intensive Care Unit, COVID-19, Enfermeros y enfermeras, Carga de trabajo, Gravedad del paciente, Unidad de Cuidados Intensivos, COVID-19

## Abstract

**Objective:**

To evaluate the workload and severity of patients in the Intensive Care Unit (ICU) with COVID-19.

**Method:**

Cross-sectional, analytical study carried out in the ICU of a private hospital. All patients over the age of 18 with a diagnosis of COVID-19 admitted from September 2020 to June 2021 were included. Workload assessed by the Nursing Activities Score (NAS), and severity by the Sequential Organ Failure Assessment. Descriptive and inferential analyses were performed.

**Results:**

217 patients were included, mostly men, mean age 62.41 years, white, obese, non-smokers and sedentary. The average NAS was 84.79. Staffing was in line with legislation and NAS. NAS was not associated with severity. Severity was associated with higher age, gender, comorbidities, sedentary lifestyle, time on mechanical ventilation, hospitalization and death.

**Conclusion:**

Workload was high and not associated with severity or outcomes. Severity was associated with demographic and clinical conditions. This study shows the importance of staff sizing, with a view to promoting safety and quality of care.

## RESUMEN

**Objetivo:**

Evaluar la carga de trabajo y la gravedad de los pacientes de la Unidad de Cuidados Intensivos (UCI) con COVID-19.

**Método:**

Estudio transversal y analítico realizado en la UCI de un hospital privado. Se incluyeron todos los pacientes mayores de 18 años con diagnóstico de COVID-19 ingresados entre septiembre de 2020 y junio de 2021. Carga de trabajo evaluada mediante la Nursing Activities Score (NAS), y gravedad mediante la valoración secuencial de fallo orgánico. Se realizaron análisis descriptivos e inferenciales.

**Resultados:**

Se incluyeron 217 pacientes, en su mayoría hombres, edad media 62,41 años, raza blanca, obesos, no fumadores y sedentarios. El NAS medio era de 84,79. Los niveles de personal se ajustaban a la legislación y al NAS. El NAS no se asoció con la gravedad. La gravedad se asoció a mayor edad, sexo, comorbilidades, sedentarismo, tiempo de ventilación mecánica, hospitalización y muerte.

**Conclusión:**

La carga de trabajo fue elevada y no se asoció a la gravedad ni a los resultados. La gravedad se asoció a las condiciones demográficas y clínicas. Este estudio muestra la importancia del dimensionamiento del personal, con vistas a promover la seguridad y la calidad de los cuidados.

## INTRODUCTION

Health systems have faced significant challenges in dealing with the increase in hospitalizations since the start of the COVID-19 pandemic, especially in Intensive Care Units (ICUs)^(1)^. Studies indicate that around 20% of those infected with SARS-CoV-2 required hospitalization, with 25% of these cases requiring intensive care, mainly due to acute respiratory syndrome^(2)^.

Several studies in China^(3)^ and Brazil^(4)^ revealed different ICU admission rates, of 5% and 31.2%, respectively. With regard to high mortality rates, one study in Italy^(5)^ showed that it was approximately 50% while in Brazil it was 35%^(4)^.

In the face of extremely severe cases, with long periods of hospitalization and a high mortality rate in the ICU, there has been a significant increase in the workload of nursing professionals. Nursing workload can be defined as the amount of time and care applied directly or indirectly to the patient, the workplace and professional development^(6)^. One of the world’s most widespread instruments for measuring nursing workload is the Nursing Activities Score (NAS). The workload is expressed as a percentage, indicating the number of hours dedicated to caring for each patient^(7)^. Studies in Belgium^(8)^ and Rio de Janeiro^(9)^ showed an increased workload for COVID-19 patients. In this context, legislation regulates the appropriate sizing of nursing professionals in the ICU^(10,11)^, to ensure quality and safe care. In the context of the pandemic, the Brazilian Federal Nursing Council (COFEN) established guidelines, increasing the number of professionals per bed in these units^(12)^.

Studies with critically ill patients often need to be based on the severity of the clinical condition, as this variable has an impact on various outcomes^(13)^. Worldwide, the Sequential Organ Failure Assessment (SOFA) has been used for this purpose^(14)^.

Intensive care nursing faces the challenge of preparing for new public health emergencies, but its goal must be to make health systems more efficient, with lower costs and greater equity for the population. In addition to maintaining the well-being of professionals, it contributes to quality of life and care^(15)^.

Against this backdrop, the objectives of the study were to evaluate nursing workload and patient severity in the ICU with COVID-19, compare the patient/professional relationship with legislation and the NAS, and associate workload with patient outcomes.

## METHOD

### Design, Period and Site

It is a cross-sectional, analytical study, guided by the STROBE (Strengthening the Reporting of Observational Studies in Epidemiology) tool, carried out from September 2020 to December 2022 at the Hospital and Maternity Hospital of the Serviço Social da Indústria do Papel, Papelão e Cortiça do Estado de São Paulo (Social Service of the Paper, Cardboard and Cork Industry of the State of São Paulo) (SEPACO). This is a private institution in the state of São Paulo, which provides care for highly complex patients. The study was carried out in the adult’s ICU for COVID-19 patients.

### Population

All patients over the age of 18 admitted to the ICU for more than 24 hours with a diagnosis of COVID-19 between September 2020 and June 2021 were included. Patients with incomplete variables of interest were excluded.

### Data Collection

The study recorded demographic and clinical variables and outcomes at the end of hospitalization.

The Sequential Organ Failure Assessment (SOFA) was assessed by the doctor on admission to the ICU. This score is made up of six systems: respiratory, assessed by the partial pressure of oxygen ratio and the fraction of inspired oxygen; hematological, through the number of platelets; hepatic, assessed by the serum bilirubin level; cardiovascular, by the presence or absence of hypotension given by the mean arterial pressure or the use of vasoactive drugs classified according to dose; neurological, analyzed by the Glasgow Coma Scale; and renal calculated by the serum creatinine level or urine output. Scores between zero and four are assigned to each of the six systems, and the scores are added together to give a total SOFA score, which can range from zero to 24 points, with the higher the score, the greater the degree of organ dysfunction^(14)^.

The workload was measured daily using the NAS by the ICU nurse, taking into account the last 24 hours of the patient’s hospitalization. The instrument consists of seven categories and 23 items: 1 - Monitoring and controls; 2 - Laboratory investigations; 3 - Medication, except vasoactive drugs; 4 - Hygiene procedures; 5 - Care of drains - All (except gastric tube); 6 - Mobilization and positioning; 7 - Support and care for relatives and patients; 8 - Administrative and managerial tasks; 9 to 11 - Ventilatory support; 12 to 15 - Cardiovascular support; 16 to 17 - Renal support; 18 - Neurological support; 19 to 21 - Metabolic support; 22 to 23 - Specific interventions inside or outside the ICU. Each NAS point is equivalent to 14.4 minutes of nursing care. The sum of the scores attributed to each category results in a score, expressed as a percentage, which represents the time spent in ICU nursing care for each patient in the last 24 hours, with a maximum total of 176.8%^(7)^. It is noteworthy that the institution offers frequent training for doctors and nurses on the application of these instruments, as it values them for care planning, which increases the reliability of the information. The data was collected by the researcher from the electronic medical records.

The number of nurses and nursing technicians per shift was recorded on a daily basis using the work schedules. In order to compare the patient-to-professional ratio in the Intensive Care Unit with the legislation (Federal Nursing Council Resolution No. 543/2017 and the Ministry of Health’s RDC No. 26) and the workload, the NAS/nurse ratio was calculated, obtained by adding the NAS of all the patients in the ICU divided by the number of nurses in the sector, the sum of the NAS over 24 hours/number of nurses, and the sum of the NAS over 24 hours/number of nursing professionals, the total ratio of patients/total nurses per day and the total ratio of patients/total professionals per day.

### Data Analysis and Statistics

The Statistical Package for Social Science (SPSS) program version 23 was used. Descriptive analysis was carried out by calculating the mean, standard deviation, median, minimum and maximum. Frequency and percentage were calculated for categorical variables. Inferential analysis was used: Spearman’s Correlation Coefficient to correlate NAS with the number of nurses, number of patients with the number of nurses, number of patients with number of nursing professionals by ICU time, NAS with SOFA, NAS and SOFA with age, Mechanic Ventilation (MV) time and ICU time, age with MV time and ICU time. Mann-Whitney test was used for association of NAS with number of nurses, number of patients with number of nurses and number of patients with number of professionals with outcomes, association of MV time, ICU time with smoking and association of age with outcomes. The chi-square test and Fisher’s exact test was applied for the association of outcomes with BMI, comorbidities and smoking, as well as the Kruskal-Wallis test for the association of MV time, ICU time with BMI and comorbidities. The significance level considered was 5% (p-value < 0.05).

### Ethical Aspects

The study was cleared by the Research Ethics Committee (CEP) of the Federal University of São Paulo, opinion number: 5.243. 290 and by the co-participating institution. The CEP granted the waiver of the Informed Consent Form (ICF), since most of the patients were admitted to the ICU seriously ill and with impaired level of consciousness, in the context of a Public Health emergency and with a high risk of contamination and spread of COVID-19. Data from medical records was used and the anonymity of the patients was guaranteed.

## RESULTS

The study population consisted of 217 patients. The majority were male (57.1%), with a mean age of 62.41 (SD:+17.18) years, white (78.8%), married (64.5%), retired/pensioners (37.8%), mostly obese (38.7%), non-smokers (96.3%), sedentary (82.9%), with a mean duration of mechanical ventilation of 8.99 (SD: +14.4) days, stayed in the ICU for an average of 16.25 (SD:+15.86) days and 64.1% were discharged from the unit (Table 1).

**Table 1:** Sociodemographic and clinical characterization of COVID-19 patients admitted to the Intensive Care Unit – São Paulo, Brazil, 2021.

Sociodemographic variables	n(%)	Clinical variables	n(%)
**Age**		Time in ICU* (total/day)	
Mean (SD)	62.41 (±17.18)	Mean (SD)	16.25 (±15.86)
Median	63	Median	11
Minimum-Maximum	23–97	Minimum-Maximum	1–89
**Sex**		**Sedentarism**	
Men	124 (57.1)	Yes	180 (82.9)
Women	93 (42.9)	No	37 (17.1)
**Race**		Time MV** (total/day)	
White	171 (78.8)	Mean (SD)	8.99 (±14.14)
Brown	37 (17.1)	Median	1
Others	9 (4.1)	Minimum-Maximum	0–89
**Marital Status**		BMI classification***	
Married	140 (64.5)	Slimness	1 (0.5)
Widowed	19 (8.8)	Normal	46 (21.2)
Single	21 (9.7)	Overweight	77 (35.5)
Separated	13 (6)	Obesity/Serious obesity	93 (42.8)
Others	24 (11.1)	**Smoker**	
**Occupation**		Yes	8 (3.7%)
Employee	70 (32.3)	No	209 (96.3%)
Retired/pensioner	82 (37.8)	**Outcome**	
Household	39 (18)	Discharge	139 (64.1)
		Deceased	78 (35.9)

*Intensive Care Unit.

**Mechanical Ventilation.

***Body Mass Index.

The daily application of the NAS in the 217 patients resulted in 3547 records, the average NAS was 84.79 (SD: +10.47), the overall description of the items marked is shown in Table 2.

**Table 2:** Frequency of items marked on the Nursing Activities Score for COVID-19 patients in the Intensive Care Unit – São Paulo, Brazil, 2021 (n = 3547).

NAS items (score)	Total (%)
**1. Monitorization and controls**	
1 a. Vital signs, calculation and recording of water balance (4.5 points)	122 (3.4)
1 b. Presence at the bedside and continuous or active observation for 2 hours or more on any shift for reasons of safety, severity or therapy, such as: NIMV, weaning, agitation, mental confusion, prone position, preparation and administration of fluids or medicines and assistance with specific procedures. (12.1 Points).	2876 (81.1)
1 c. Presence at the bedside and continuous or active observation for 4 hours or more on any shift due to safety, severity or therapy. (19.6 Points)	549 (15.5)
**2. Lab tests: Biochemistry and microbiology. (0 and 4.3 Points)**	
Present	3530 (99.5)
Absent	17 (0.5)
**3. Medication, excepted vasoactive drugs (0 and 5.6 Points)**	
Present	3534 (99.6)
Absent	13 (0.4)
**4. Hygiene procedures**	
4 a. Normal (4.1 Points)	1680 (47.4)
4 b. Carrying out hygiene procedures lasting more than 2 hours in any shift (16.5 Points)	1728 (48.7)
4 c. Carrying out hygiene procedures lasting more than 4 hours in any one shift. (20 Points)	139 (3.9)
**5. Care of drains. All except gastric tube (0 and 1.8 points)**	
Present	402 (11.3)
Absent	3145 (88.7)
**6. Mobilization and positioning**	
6 a. Performing the procedure(s) up to three times in 24 hours. (5.5 Points)	362 (10.2)
6 b. Performing the procedure(s) more than three times in 24 hours or with 2 nurses at any frequency. (12.4 Points)	2877 (81.1)
6 c. Performing the procedure(s) with 3 or more nurses at any frequency. (17.0 Points)	308 (8.7)
**7. Support and care for relatives and patients**	
7 a. Support and care for family members and patients who require exclusive dedication for around 1 hour on any shift, such as: explaining medical conditions, dealing with difficult family circumstances. (4.0 Points)	3458 (97.5)
7 b. Support and care for family members and patients who require exclusive dedication for 3 hours or more on any shift, such as: death, special circumstances (e.g. large numbers of family members, language problems and hostile families). (32.0 Points).	89 (2.5)
**8. Administrative and managerial tasks**	
8 a. Carrying out routine tasks such as: clinical data procedures, requesting examinations and exchanging professional information (e.g. on call and clinical visits). (4.2 Points)	2742 (77.3)
8 b. Carrying out administrative and managerial tasks that require full dedication for around 2 hours on any given shift, such as: research activities, application of protocols, admission and discharge procedures. (23.2 Points)	799 (22.5)
8 c. Carrying out administrative and managerial tasks that require full dedication for around 4 hours or more on any given shift, such as: death and organ donation procedures, coordinated with other disciplines. (30.0 Points)	6 (0.2)
**9. Respiratory support (0 and 1.4 points)**	
Present	3213 (90.6)
Absent	334 (9.4)
**10. Care of artificial airways (0 and 1.8 points)**	
Present	2083 (58.7)
Absent	1464 (41.3)
**11. Treatment to improve pulmonary function (0 and 4.4 points)**	
Present	3291 (92.8)
Absent	256 (7.2)
**12. Vasoactive drugs (0 and 1.2 points)**	
Present	1491 (42)
Absent	2056 (58)
**13. Intravenous replacement of major fluid losses (0 and 2.5 points)**	
Present	1242 (35)
Absent	2305 (65)
**14. Left atrium monitoring. Pulmonary artery catheter with or without cardiac output measurements. (0 and 1.7 Points)**	
Present	18 (0.5)
Absent	3529 (99.5)
**15. Cardiopulmonary resuscitation in the last 24 hours. Excludes precordial thump. (0 and 1.7 Points)**	
Present	11 (0.3)
Absent	3536 (99.7)
**16. Hemofiltration techniques. Dialysis techniques (0 and 7.7 points)**	
Present	726 (20.5)
Absent	2821 (79.5)
**17. Quantitative measurement of urine output (0 and 7.0 points)**	
Present	3194 (90)
Absent	353 (10)
**18. Intracranial pressure measurements (0 and 1.6 points)**	
Present	18 (0.5)
Absent	3529 (99.5)
**19. Treatment of metabolic acidosis/alkalosis. (0 and 1.3 Points)**	
Present	988 (27.9)
Absent	2559 (72.1)
**20. Total Parenteral Nutrition (0 and 2.8 points)**	
Present	89 (2.5)
Absent	3458 (97.5)
**21. Enteral feeding by gastric tube or other gastrointestinal route (0 and 1.3 points)**	
Present	2145 (60.5)
Absent	1402 (39.5)
**22. Specific intervention(s) in the Intensive Care Unit.**	
Present	3213 (90.6)
Absent	334 (9.4)
**23. Specific interventions outside the Intensive Care Unit (0 and 1.9 points)**	
Present	514 (14.5)
Absent	3033 (85.5)

The mean number of patients/nurses was 3.27 (SD:+2.19), the mean number of patients/professionals was 1 (SD:+0.62), while the mean number of NAS/nurses was 14.63 (SD:+5.12) and the mean number of NAS/professionals was 4.51 (SD:+0.6).

There was no significant association between the discharge and decease outcomes and the ratios: average number of NAS per nurse (p = 0.6233), average number of patients per average number of nurses (p = 0.4884) and average number of patients per number of professionals (p = 0.6377).

There was no association between NAS and SOFA, age, MV time and ICU time (p > 0.05). However, higher SOFA scores were associated with older patients, longer MV times and longer ICU stays (Table 3).

**Table 3:** Association between Nursing Activities Score and Sequential Organ Failure Assessment with age, duration of mechanical ventilation and length of stay in the Intensive Care Unit – São Paulo, Brazil, 2021 (n = 217).

		SOFA	Age	Mechanical ventilation time	Time in ICU**
**NAS**	p-value	0.6415	0.4524	0.7067	0.9784*
**SOFA**	p-value	-	0.0007	0.0001	0.0001

*Spearman’s Correlation Coefficient.

**Intensive Care Unit.

There was no association between the variables sex (p = 0.1129), race (p = 0.2126), BMI (p = 0.1890), sedentary lifestyle (p = 0.8531), smoking (p = 0.1686) number of comorbidities (p = 0.1015) and outcome (p = 0.5743) with NAS.

The association between SOFA and sociodemographic and clinical variables showed that male patients had higher scores than females, sedentary patients had higher SOFA scores than non-sedentary patients and patients who died had higher scores than those who were discharged (Table 4).

**Table 4:** Association between Sequential Organ Failure Assessment severity index and sociodemographic and clinical variables – São Paulo, Brazil, 2021 (n = 217).

Variables	Sequential organ failure Assessment				p-value
	N	Mean (SD)	Median	Minimum-maximum	
**Sex**					
Men	124	3.15 (2.37)	3	0–17	0.0141*
Women	93	2.46 (1.99)	2	0–11	
**Body Mass Index**					
Slimness/Normal	47	3.3 (3.3)	3	0–17	0.9562**
Overweight	77	2.74 (1.86)	3	0–9	
Obesity/Serious Obesity	93	2.72 (1.81)	3	0–10	
**Race**					
White	171	2.96 (2.29)	3	0–17	0.1384*
Non-white	46	2.43 (2)	2	0–9	
**Smoker**					
Yes	8	3.13 (1.55)	3.5	0–5	0.3324*
No	209	2.84 (2.26)	3	0–17	
**Sedentarism**					
Yes	180	3.02 (2.05)	3	0–13	0.0002*
No	37	2.03 (2.86)	2	0–17	
**Comorbidities**					
None	41	3.02 (2.91)	2	0–17	0.0529**
1–2	101	2.45 (1.69)	2	0–9	
3–4	54	3.22 (2.15)	3	0–11	
5 or more	21	3.52 (2.94)	4	0–13	
**Outcome**					
Discharge	139	2.32 (2.03)	2	0–17	<0.0001*
Decease	78	3.81 (2.27)	3	0–13	

*Mann-Whitney test.

**Kruskal-Wallis test.

The time length of mechanical ventilation was not associated with age (p = 1877), BMI (p = 0.3027), smoking (p = 0.6237) or the number of comorbidities (p = 0.2737). Length of stay in the ICU was only associated with age; the older the patient, the longer the length of stay (p < 0.0170).

The outcomes decease and discharge were associated with age and number of comorbidities, where patients who died had a higher average age than those who were discharged and patients with five or more comorbidities had a higher proportion of deaths than those with fewer comorbidities (Table 5).

**Table 5:** Association of discharge and decease outcomes with age and clinical variables – São Paulo, Brazil, 2021 (n = 217).

	Outcomes		p-value
	Discharge n (%)	Decease n (%)	
**Age**			
Mean (±SD)	58.27 (±17.17)	69.77 (±14.61)	<0.0001*
Median	60	73.5	
Minimum-Maximum	23–92	24–97	
**Body Mass Index**			
Slimness/Normal	32 (68.1)	15 (31.9)	0.7246**
Overweight	47 (61)	30 (39)	
Obesity/Serious Obesity	60 (64.5)	33 (35.5)	
**Smoker**			
Yes	4 (50)	4 (50)	0.4622***
No	135 (64.6)	74 (35.4)	
**Comorbidities**			
None	32 (78)	9 (22)	0.0421**
1–2	66 (65.3)	35 (34.7)	
3–4	32 (59.3)	22 (40.7)	
5 or more	9 (42.9)	12 (57.1)	

*Mann-Whitney test.

**Chi-square test.

***Fisher’s exact test.

## DISCUSSION

Studies carried out on COVID-19 patients who required ICU admission showed sociodemographic characteristics similar to the findings of this study, where the majority were men, elderly, obese and had comorbidities, as well as similar times of MV and ICU admission^(1,4-5)^. A study carried out using hospitalization data from the Ministry of Health, deaths provided by the Civil Registry and population data from the Brazilian Institute of Geography and Statistics (IBGE), with the aim of identifying differences in mortality and hospitalization by gender and age in COVID-19 patients, showed that men had a greater predisposition to not following isolation protocols, thus increasing the chance of contamination and that the elderly, due to senescence and the presence of chronic diseases, common at this age, increased the chances of hospitalization due to the worsening of the disease^(16)^.

Measuring the workload of the nursing team, using the NAS, is very important for adjusting the number of professionals per patient, which is associated with lower numbers of adverse events, lower numbers of healthcare-related infections and hospital mortality. In this context, it is directly related to the quality of care, guaranteeing safety for patients and the care team^(8)^.

The average NAS of the population in this study was 84.79, which is equivalent to 20.34 hours of assistance from the nursing team. Studies carried out to demonstrate the impact of the pandemic on nursing workload in Belgium, Italy and the Netherlands found average NAS of 92, 84 and 55 respectively^(17)^. In Brazil, a study of cancer patients with COVID-19 found an average NAS of 110^(9)^ and another of ICU patients with COVID-19 found an average of 86^(18)^. In this context, studies have shown that patients with COVID-19 had higher mean scores when compared to patients without a diagnosis of the disease. The analysis of the items marked on the NAS in this study allowed us to conclude that most patients required more hours of monitoring than usual, the use of a greater number of professionals to carry out hygiene procedures and that most of them required interventions within the ICU, in addition to the frequent need for MV, renal replacement therapy and Extra Corporeal Membrane Oxygenation (ECMO), which may justify the increase in NAS in this population.

With regard to the main activities assessed through NAS, the results are similar to those found in two other studies^(8,19)^. The study carried out in Belgium with 95 patients and another carried out in the Netherlands with 218 patients, both showed that COVID-19 patients required more hours of monitoring, due to the severity of the patients and that this time may also have been increased by the need for complex clothing and personal protective equipment. They also showed that a greater number of professionals were needed for hygiene procedures and decubitus changes, mainly due to the prone position, the decubitus used as the gold standard for the treatment of patients with severe respiratory failure. Another similar aspect was the care given to family members, due to the high mortality rate and limitations on visits during the pandemic. This explains the complexity of caring for patients with COVID-19.

No study was found with the participation of COVID-19 patients that carried out the association between the number of nurses/number of patients, number of professionals/number of patients and NAS/number of nurses, however a Dutch study with the inclusion of data on the workload of 29.445 patients collected in ICUs from January 2016 to January 2018 demonstrated the association between workload and hospital mortality^(20)^. In this study, the sizing of the nursing team was in line with that proposed by the Ministry of Health, COFEN and the NAS, i.e. there was a model scenario for safe and quality care practice^(10–12)^. In this context of staff sizing, there was no association between this relationship and MV time, length of ICU stay and mortality.

In this study, nursing workload was not correlated with the severity of the subjects. A different result was found in a study which showed a moderate positive relationship between NAS and SOFA^(21)^. The divergence in results can be explained by the fact that SOFA was only assessed on admission, unlike NAS, which was carried out daily; perhaps more frequent collection of the severity index is necessary to improve the correlation between these variables.

Higher SOFA scores were found in older patients, who spent longer on MV and in the ICU. A study carried out in the United States, with the participation of five ICUs and the inclusion of 2320 patients diagnosed with COVID-19, also found an association between SOFA and age^(22)^. Studies have shown a relationship between age and greater patient severity, i.e. requiring longer ventilation times and longer hospital stays^(1–5)^.

A systematic review carried out with the aim of identifying prognostic factors for severity and mortality in COVID-19 patients, including 207 studies, showed that male gender was a predictor of severity^(23)^. Studies point to social and biological issues as probable determining factors for this, since there is a higher proportion of male smokers, a greater predisposition of males to neglect their health, and there is a difference in the immune and cellular response between the sexes, which may justify higher SOFA scores among men^(16.23)^.

In this study, a sedentary lifestyle was associated with greater severity, a result that is in line with those of a cohort of 387.109 adults in the United Kingdom, which showed a relationship between physical activity and COVID-19 severity. Individuals with a worse lifestyle had a four times greater risk of worsening the disease, as there is evidence that physical activity has beneficial effects on the immune system, such as anti-inflammatory effects and a better adaptive immune response^(24)^.

In this study, higher SOFA scores were found among patients who died. Higher SOFA scores indicate severe or multiple organ dysfunction, conditions which are commonly found in patients who develop the severe form of the disease. Although it has not been proposed to predict mortality, studies have shown that an increase in the score during the first 96 hours in the ICU or during hospitalization has been associated with a higher risk of death^(14)^. A study carried out in the United States with 320 COVID-19 patients who developed severe respiratory symptoms showed a correlation between the score and mortality, where a score between 0 and 1 was associated with a 100% chance of survival and scores greater than 11 with mortality in 100% of cases^(25)^. In this context, this score can be used as a prognostic parameter for patients affected by COVID-19.

The association between length of stay and age has been demonstrated in several studies of patients with COVID-19, where those with older age stayed in hospital longer^(1–5)^. The increase in ICU admissions among the elderly is an expected phenomenon due to population aging and the presence of chronic degenerative diseases, commonly found with advancing age. The elderly are more debilitated, have fewer physiological reserves, and the decline and dysregulation of immune function, known as immunosenescence, results in a less efficient response to infection^(26,27,28)^. Thus, those who are older have a less efficient immune response and may require more care and resources, resulting in a prolonged recovery time, making it necessary to stay longer in the units, justifying this association.

A higher proportion of deaths was found among those who were older and had a greater number of comorbidities. Worldwide data shows more than 3.4 million deaths from COVID-19 by March 2023, 2.4 million of which were people over 65(28). An international cohort with the participation of 52 countries and the inclusion of 600.000 patients showed that an increase of 10 years in the subject’s age increased the risk of death from COVID-19 1.5 times^(28)^. Ageing has been associated with an increase in specific biomarkers reflecting greater endothelial activation, activation of the coagulation system, inflammatory cytokines and, therefore, organ damage^(29)^. With regard to comorbidities, the presence of virus receptors in places such as the lungs, heart and gastrointestinal system, and the increase in the number of these receptors in some diseases such as asthma and diabetes, increases the vulnerability to contamination by the virus. This can lead to decompensation of chronic diseases, resulting in multiple organ dysfunction and death^(29,30)^. Thus, age and comorbidities have been associated with unfavorable outcomes.

We have thus made progress in assessing the impact of the pandemic on the severity of patients and consequently on the nursing workload, demonstrating the main demands of these patients in relation to nursing care. In this context, we highlight that the NAS was an essential tool for the effective management of ICU care, ensuring quality care for critically ill patients, in a pandemic scenario, which brought several challenges for the care team with the changes in the epidemiological profile. It also helps to prevent professional overload, contributing to the health and well-being of nursing professionals, as its use promotes a balance between work demand and available resources, favoring a more efficient and safer health system. The fact that it was carried out in a single center limited the number of participants, and the fact that the SOFA was only assessed on patient admission can be a limitation when associated with the workload, which was assessed on a daily basis.

## CONCLUSION

The workload of the subjects in this study was high, requiring 20.34 hours of nursing care. The severity of the patients meant that more hours of monitoring and a greater number of professionals were used for hygiene and decubitus change procedures. There was no association between workload and the outcomes time on mechanical ventilation, length of ICU stays, discharge and death. No association was found between NAS and SOFA; perhaps more frequent calculations of the severity index give different results. Patient severity was associated with demographic and clinical conditions, such as older age, more comorbidities, longer duration of mechanical ventilation and ICU stay, and higher mortality. Identifying the nursing workload with a view to correct sizing contributes to the quality of care and safety for patients and professionals.

## References

[1] Wang Z, Liu Y, Wei L, Ji JS, Liu Y, Liu R (2022). What are the risk factors of hospital length of stay in the novel
coronavirus pneumonia (COVID-19) patients? A survival analysis in South west
China. PLoS One.

[2] Rees EM, Nightingale ES, Jafari Y, Waterlow NR, Clifford S, Pearson CAB (2020). COVID-19 length of hospital stay: a systematic review and data
synthesis. BMC Med.

[3] Guan WJ, Ni ZY, Hu Y, Liang WH, Ou CQ, He JX (2020). Clinical characteristics of Coronavirus Disease 2019 in
China. N Engl J Med.

[4] Wolf JM, Petek H, Maccari JG, Nasi LA (2022). COVID-19 pandemic in Southern Brazil: hospitalizations, intensive
care unit admissions, lethality rates, and length of stay between March 2020
and April 2022. J Med Virol.

[5] Grasselli G, Greco M, Zanella A, Albano G, Antonelli M, Bellani G (2020). COVID-19 Lombardy ICU Network. Risk factors associated with
mortality among patients with COVID-19 in intensive care units in Lombardy,
Italy. JAMA Intern Med.

[6] Alghamdi MG (2016). Nursing workload: a concept analysis.. J Nurs Manag.

[7] Bruyneel A, Tack J, Droguet M, Maes J, Wittebole X, Miranda DR (2019). Measuring the nursing workload in intensive care with the Nursing
Activities Score (NAS): a prospective study in 16 hospitals in
Belgium. J Crit Care.

[8] Bruyneel A, Gallani MC, Tack J, d’Hondt A, Canipel S, Franck S (2021). Impact of COVID-19 on nursing time in intensive care units in
Belgium. Intensive Crit Care Nurs.

[9] Lima VCGS, Pimentel NBL, Oliveira AM, Andrade KBS, Santos MLSC, Fuly PCS (2023). Carga de trabalho da enfermagem de terapia intensiva oncológica
na pandemia da COVID-19: coorte retrospectiva. Rev Gaúcha Enferm.

[10] Brasil. (2012). Ministério da Saúde. Agência Nacional de Vigilância Sanitária
(ANVISA). Resolução da Diretoria Colegiada – RDC n° 26, de 11 de maio de
2012, dispõe sobre Requisitos mínimos para funcionamento de Unidades de
Terapia Intensiva e dá outras providências [Internet]. Diário Oficial da União, Poder Executivo, Brasília;.

[11] Conselho Federal de Enfermagem (COFEN). Resolução n°
543/2017. (2017). Atualiza e estabelece parâmetros para o dimensionamento do quadro
de profissionais de enfermagem nos serviços locais em que são realizadas
atividades de enfermagem. [Internet]. Brasília;.

[12] Conselho Federal de Enfermagem (COFEN). (2020). Parecer Normativo nº 002/2020/COFEN – exclusivo para vigência da
pandemia covid-19. Gt dimensionamento de pessoal. Parâmetros mínimos de
profissionais de Enfermagem para atendimento aos pacientes acometidos pela
COVID-19. [Internet]. Brasília;.

[13] Moreno R, Rhodes A, Piquilloud L, Hernandez G, Takala J, Gershengorn HB (2023). The Sequential Organ Failure Assessment (SOFA) Score: has the
time come for an update?. Crit Care.

[14] 14.Ferreira FL, Bota DP, Bross A, Mélot C, Vincent JL (2001). Serial evaluation of the SOFA score to predict outcome in
critically ill patients. JAMA.

[15] Hermann B, Benghanem S, Jouan Y, Lafarge A, Beurton A (2023). The positive impact of COVID-19 on critical care: from
unprecedented challenges to transformative changes, from the perspective of
young intensivists. Ann Intensive Care.

[16] Souza LG, Randow R, Siviero PCL (2020). Reflexões em tempos de COVID-19: diferenciais por sexo e
idade. Com. Ciênc Saúde (Porto Alegre).

[17] Bruyneel A, Lucchini A, Hoogendoorn M (2022). Impact of COVID-19 on nursing workload as measured with the
Nursing Activities Score in intensive care. Intensive Crit Care Nurs.

[18] Buffon MR, Severo IM, Barcellos RA, Azzolin KO, Lucena AF (2022). Critically ill COVID-19 patients: a sociodemographic and clinical
profile and associations between variables and workload. Rev Bras Enferm.

[19] Hoogendoorn ME, Brinkman S, Bosman RJ, Haringman J, de Keizer NF, Spijkstra JJ (2021). The impact of COVID-19 on nursing workload and planning of
nursing staff on the Intensive Care: a prospective descriptive multicenter
study. Int J Nurs Stud.

[20] Margadant C, Wortel S, Hoogendoorn M, Bosman R, Spijkstra JJ, Brinkman S (2020). The Nursing Activities Score Per Nurse Ratio Is Associated With
In-Hospital Mortality, Whereas the Patients Per Nurse Ratio Is
Not. Crit Care Med.

[21] Sirqueira EMP, Ribeiro MD, Souza RCS, Machado FS, Diccini S (2015). Correlação entre carga de trabalho de enfermagem e gravidade dos
pacientes críticos gerais, neurológicos e cardiológicos. Esc Anna Nery.

[22] Tolchin B, Oladele C, Galusha D, Kashyap N, Showstark M, Bonito J (2021). Racial disparities in the SOFA score among patients hospitalized
with COVID-19. PLoS One.

[23] Izcovich A, Ragusa MA, Tortosa F, Lavena Marzio MA, Agnoletti C, Bengolea A (2020). Prognostic factors for severity and mortality in patients
infected with COVID-19: a systematic review. PLoS One.

[24] Hamer M, Kivimäki M, Gale CR, Batty GD (2020). Lifestyle risk factors, inflammatory mechanisms, and COVID-19
hospitalization: a community-based cohort study of 387.109 adults in
UK. Brain Behav Immun.

[25] Fayed M, Patel N, Angappan S, Nowak K, Vasconcelos Torres F, Penning DH (2022). Sequential Organ Failure Assessment (SOFA) score and mortality
prediction in patients with severe respiratory distress secondary to
COVID-19. Cureus.

[26] Chen Y, Klein SL, Garibaldi BT, Li H, Wu C, Osevala NM (2021). Aging in COVID-19: vulnerability, immunity and
intervention. Ageing Res Rev.

[27] Veiga VC, Cavalcanti AB (2023). Age, host response, and mortality in COVID-19. Eur Respir J.

[28] Kartsonaki C, Baillie JK, Barrio NG, Baruch J, Beane A, Blumberg L (2023). Characteristics and outcomes of an international cohort of 600
000 hospitalized patients with COVID-19. Int J Epidemiol.

[29] Ejaz H, Alsrhani A, Zafar A, Javed H, Junaid K, Abdalla AE (2020). COVID-19 and comorbidities: deleterious impact on infected
patients. J Infect Public Health.

[30] Djaharuddin I, Munawwarah S, Nurulita A, Ilyas M, Tabri NA, Lihawa N (2021). Comorbidities and mortality in COVID-19 patients. Gac Sanit.

