# Supplementary material for: Nursing workload and severity of COVID-19 patients in the Intensive Care Unit
Source: Rev Esc Enferm USP. 2024 Aug 23;58:e20240107. doi: 10.1590/1980-220X-REEUSP-2024-0107en (PMC11349331; doi:10.1590/1980-220X-REEUSP-2024-0107en)
Supplement: Translation

# Translation: Carga de trabalho de Enfermagem e gravidade de pacientes com COVID-19 na Unidade de Terapia Intensiva

**DOI:** 10.1590/1980-220X-REEUSP-2024-0107pt

**Authors:** Wesley Cajaiba Santos, Maria Carolina Barbosa Teixeira Lopes, Cassia Regina Vancini-Campanharo, Daniela Boschetti, Sirlei Oliveira da Silva Dias, Meire Cristina Novelli e Castro, Luis Humberto Vieri Piacezzi, Ruth Ester Assayag Batista

**Affiliations:** 1 Universidade Federal de São Paulo, Escola Paulista de Enfermagem, São Paulo, SP, Brasil.; 2 Hospital e Maternidade do SEPACO, Serviço Social da Indústria do Papel, Papelão e Cortiça do Estado de São Paulo, São Paulo, SP, Brasil.; 3 Faculdade de Medicina da Universidade Estadual Paulista Júlio de Mesquita Filho, Departamento de Enfermagem, Botucatu, SP, Brasil.

**Keywords:** Enfermeiras e Enfermeiros, Carga de Trabalho, Gravidade do Paciente, Unidade de Terapia Intensiva, COVID-19

## Abstract

**Objetivos:**

Avaliar carga de trabalho e gravidade dos pacientes na Unidade de Terapia Intensiva (UTI) com COVID-19.

**Método:**

Estudo transversal, analítico realizado na UTI em hospital privado. Incluídos todos os pacientes maiores de 18 anos, com diagnóstico de COVID-19 admitidos de setembro de 2020 a junho de 2021. Carga de trabalho avaliado pelo Nursing Activities Score (NAS), e gravidade pelo Sequential Organ Failure Assessment. Realizado análises descritiva e inferencial.

**Resultados:**

Incluídos 217 pacientes, maioria homens, média de idade 62,41 anos, brancos, obesos, não tabagistas e sedentários. A média do NAS foi 84,79. O dimensionamento de pessoal estava em concordância com legislação e NAS. O NAS não foi associado a gravidade. Houve associação da gravidade com maior idade, sexo, comorbidades, sedentarismo, tempo de ventilação mecânica, internação e óbito.

**Conclusão:**

A carga de trabalho foi alta e não associada a gravidade e desfechos. A gravidade foi associada às condições demográficas e clínicas. Este estudo mostra a importância do dimensionamento de pessoal, com vistas à promoção da segurança e qualidade assistencial.

## INTRODUÇÃO

Desde o início da pandemia de COVID-19, os sistemas de saúde enfrentaram desafios significativos para lidar com o aumento das internações, especialmente nas Unidades de Terapia Intensiva (UTI)^(1)^. Estudos indicam que cerca de 20% dos infectados pelo SARS-CoV-2 necessitaram de hospitalização, com 25% desses casos demandando cuidados intensivos, devido, principalmente, a síndrome respiratória aguda^(2)^.

Pesquisas na China^(3)^ e no Brasil^(4)^ revelaram diferentes taxas de admissão na UTI, de 5% e 31,2%, respectivamente. Em relação as altas taxas de mortalidade, no estudo na Itália^(5)^ foi de aproximadamente 50% enquanto no Brasil foi de 35%^(4)^.

Diante de pacientes extremamente graves, com prolongados períodos de internação e alta taxa de mortalidade na UTI, houve aumento expressivo na carga de trabalho dos profissionais de enfermagem. A carga de trabalho de enfermagem pode ser definida como a quantidade de tempo e cuidado aplicado de forma direta ou indiretamente, para o paciente, local de trabalho e para o desenvolvimento profissional^(6)^. Um dos instrumentos mais difundido mundialmente para medição da carga de trabalho de enfermagem é o Nursing Activities Score (NAS), a carga de trabalho é expressa por porcentagem, indicando a quantidade de horas dedicadas ao cuidado de cada paciente^(7)^. Estudo na Bélgica^(8)^ e no Rio de Janeiro^(9)^, evidenciaram a aumento da carga de trabalho nos pacientes com COVID-19. Neste contexto, legislações regulamentam o dimensionamento adequado dos profissionais de enfermagem na UTI^(10,11)^, para garantir assistência de qualidade e segura. No contexto da pandemia, o Conselho Federal de Enfermagem (COFEN) estabeleceu diretrizes, aumentando o número de profissionais por leito nessas unidades^(12)^.

Estudos com pacientes críticos, frequentemente, precisam ter como balizador a gravidade do quadro clínico, pois esta variável impacta em diversos desfechos^(13)^. Mundialmente, o Sequential Organ Failure Assessment (SOFA), tem sido utilizado com esse objetivo^(14)^.

A enfermagem intensiva tem como desafio preparar-se para novas emergências em saúde pública, entretanto, deve ter como meta a eficiência dos sistemas de saúde com menor custo e maior equidade para a população. Além da manutenção do bem-estar dos profissionais, contribuindo para a qualidade de vida e a assistencial^(15)^.

Diante disso, os objetivos do estudo são avaliar a carga de trabalho de Enfermagem e a gravidade dos pacientes na UTI com COVID-19, comparar a relação paciente/profissional com a legislação e o NAS, e associar a carga de trabalho com os desfechos dos pacientes.

## MÉTODO

### Desenho, Período E Local

Estudo transversal, analítico, norteado pela ferramenta STROBE (Strengthening the Reporting of Observational Studies in Epidemiology), realizado de setembro de 2020 a dezembro de 2022 no Hospital e Maternidade do Serviço Social da Indústria do Papel, Papelão e Cortiça do Estado de São Paulo (SEPACO). Instituição privada do Estado de São Paulo, São Paulo, que presta assistência a pacientes de alta complexidade. O estudo foi realizado na UTI para adultos destinada a internação de pacientes com COVID-19.

### População

Foram incluídos todos os pacientes, maiores de 18 anos, internados na UTI, por mais de 24 horas, com diagnóstico de COVID-19, durante o mês de setembro de 2020 a junho de 2021. Foram excluídos os pacientes com incompletude das variáveis de interesse.

### Coleta de Dados

Foram registradas as variáveis demográficas e clínicas e desfechos ao final da internação.

O Sequential Organ Failure Assessment (SOFA) foi avaliado pelo médico na admissão dos pacientes na UTI. Esse escore é composto por seis sistemas: respiratório, avaliado pela relação pressão parcial de oxigênio e a fração inspirada de oxigênio; hematológico, por meio do número de plaquetas; hepático, avaliado pelo nível sérico de bilirrubina; cardiovascular, pela presença ou não de hipotensão dada pela pressão arterial média ou uso de droga vasoativa classificada conforme a dose; neurológico, analisado pela Escala de Coma de Glasgow; e renal calculado pelo nível sérico de creatinina ou débito urinário. São atribuídos escores entre zero e quatro a cada um dos seis sistemas, os escores são somados para proporcionar um escore SOFA total, que pode variar de zero a 24 pontos, sendo que quanto maior a pontuação, maior o grau de disfunção orgânica^(14)^.

A carga de trabalho foi realizada, diariamente, por meio do NAS, pelo enfermeiro da UTI, considerando as últimas 24 horas de internação do paciente. O instrumento é composto por sete categorias e 23 itens, são eles: 1 - Monitorização e controles; 2 - Investigações laboratoriais; 3 - Medicação, exceto drogas vasoativas; 4 - Procedimentos de higiene; 5 - Cuidados com drenos – Todos (exceto sonda gástrica); 6 - Mobilização e posicionamento; 7 - Suporte e cuidados aos familiares e pacientes; 8 - Tarefas administrativas e gerenciais; 9 ao 11 - Suporte ventilatório; 12 ao 15 - Suporte cardiovascular; 16 ao 17 - Suporte renal; 18 - Suporte neurológico; 19 ao 21 - Suporte metabólico; 22 ao 23 - Intervenções específicas dentro ou fora da UTI. Cada ponto do NAS equivale a 14,4 minutos de cuidados de enfermagem. A soma das pontuações atribuídas a cada categoria resulta em escore, expresso em porcentagem, que representa o tempo dispensado, em assistência de enfermagem na UTI, a cada paciente, nas últimas 24 horas, sendo seu total máximo 176,8%^(7)^. Importante destacar que a instituição oferece capacitações frequentes para médicos e enfermeiros sobre a aplicação desses instrumentos, pois os valoriza para o planejamento assistencial, o que aumenta a confiabilidade das informações. Os dados foram coletados pelo pesquisador no prontuário eletrônico.

Diariamente, foi registrado número de enfermeiros e técnicos de enfermagem por plantão, por meio das escalas de trabalho. Para comparar a relação paciente/profissional da Unidade de Terapia Intensiva com a legislação (Resolução COFEN n° 543/2017 e a RDC n° 26 do Ministério da Saúde) e a carga de trabalho foi calculado a relação NAS/enfermeiro, obtido pela soma do NAS de todos os pacientes presentes na UTI dividido pelo número de enfermeiros presentes no setor, realizando a soma do NAS em 24h/número de enfermeiros, e soma do NAS em 24h/número de profissionais de enfermagem, a razão total de pacientes/total de enfermeiros por dia e a razão total de pacientes/total de profissionais por dia.

### Análise dos dados e Estatística

Foi utilizado o programa Statistical Package for Social Science (SPSS), versão 23. A análise descritiva foi realizada por meio dos cálculos de média, desvio padrão, mediana, mínimo e máximo. Para as variáveis categóricas, calcularam-se frequência e percentagem. Para análise inferencial utilizou-se: Coeficiente de Correlação de Spearman para correlacionar NAS com o número de enfermeiros, número de pacientes com o número de enfermeiros, número de pacientes com número de profissionais de enfermagem por tempo de UTI, NAS com SOFA, NAS e SOFA com idade, tempo de Ventilação Mecânica (VM) e tempo de UTI, idade com tempo de VM e tempo de UTI. O teste de Mann-Whitney para associação do NAS com o número de enfermeiros, número de pacientes com o número de enfermeiros e número de pacientes com número de profissionais com os desfechos, a associação do tempo de VM, tempo de UTI com tabagismo e a associação da idade com desfechos. O Teste de Qui-Quadrado e Teste Exato de Fisher para associação dos desfechos com Índice de Massa Corporal (IMC), comorbidades e tabagismo. E o teste de Kruskal-Wallis para associação do tempo de VM, tempo de UTI com IMC e comorbidades. O nível de significância considerado foi de 5% (valor de p < 0,05).

### Aspectos Éticos

Estudo foi aprovado pelo Comitê de Ética em Pesquisa (CEP) da Universidade Federal de São Paulo, parecer número: 5.243. 290 e pela instituição coparticipante. Foi concedida, pelo CEP, a dispensa do Termo de Consentimento Livre e Esclarecido (TCLE), visto que a maioria dos pacientes estavam internados na UTI gravemente enfermos e com prejuízo do nível de consciência, estava na vigência de uma emergência em Saúde Pública e com alto risco de contaminação e disseminação da COVID-19. Foram utilizados os dados de prontuários e garantido o anonimato dos pacientes.

## RESULTADOS

A população do estudo foi composta por 217 pacientes. A maioria eram do sexo masculino (57,1%), com média de idade 62,41 (DP:±17,18) anos, brancos (78,8%), casados (64,5%), e aposentados/pensionistas (37,8%), a maioria obesos (38,7%), não tabagistas (96,3%), sedentários (82,9%), apresentaram tempo médio de ventilação mecânica de 8,99 (DP:±14,4) dias, permaneceram internados na UTI em média 16,25 (DP:±15,86) dias e 64,1% tiveram alta da unidade (Tabela 1).

**Tabela 1:** Caracterização sociodemográfica e clínica de pacientes com COVID-19 internados na Unidade de Terapia Intensiva – São Paulo, Brasil, 2021.

Variáveis Sociodemográficas	n(%)	Variáveis Clínicas	n(%)
**Idade**		Tempo UTI* (total/dia)	
Média (DP)	62,41 (**+**17,18)	Média (DP)	16,25 (**+**15,86)
Mediana	63	Mediana	11
Mínimo-Máximo	23-97	Mínimo-Máximo	1-89
**Sexo**		**Sedentarismo**	
Masculino	124 (57,1)	Sim	180 (82,9)
Feminino	93 (42,9)	Não	37 (17,1)
**Cor**		Tempo VM** (total/dia)	
Branco	171 (78,8)	Média (DP)	8,99 (**+**14,14)
Parda	37 (17,1)	Mediana	1
Outros	9 (4,1)	Mínimo-Máximo	0-89
**Estado Civil**		Classificação IMC***	
Casado	140 (64,5)	Magreza	1 (0,5)
Viúvo	19 (8,8)	Normal	46 (21,2)
Solteiro	21 (9,7)	Sobrepeso	77 (35,5)
Separado	13 (6)	Obesidade/Obesidade grave	93 (42,8)
Outros	24 (11,1)	**Tabagista**	
**Ocupação**		Sim	8 (3,7%)
Empregado	70 (32,3)	Não	209 (96,3%)
Aposentado/pensionista	82 (37,8)	**Desfecho**	
Do lar	39 (18)	Alta	139 (64,1)
		Óbito	78 (35,9)

*Unidade de Terapia Intensiva.

**Ventilação Mecânica.

***Índice de Massa Corporal.

A aplicação diária do NAS nos 217 pacientes resultou em 3547 registros, o NAS médio foi de 84,79 (DP:±10,47), a descrição global dos itens assinalados encontra-se na Tabela 2.

**Tabela 2:** Frequência dos itens assinalados do Nursing Activities Score dos pacientes com COVID-19 na Unidade de Terapia Intensiva - São Paulo, Brasil, 2021 (n = 3547).

Itens do NAS (pontuação)	Total (%)
**1. Monitorização e controles**	
1 a. Sinais Vitais, cálculo e registro do balanço hídrico (4,5 Pontos)	122 (3,4)
1 b. Presença à beira do leito e observação contínua ou ativa por 2 horas ou mais em algum plantão por razão de segurança, gravidade ou terapia, tais como: VMNI, desmame, agitação, confusão mental, posição prona, preparo e administração defluídos ou medicamentos e auxílio em procedimentos específicos. (12,1 Pontos).	2876 (81,1)
1 c. Presença à beira do leito e observação contínua ou ativa por 4 horas ou mais em algum plantão por razão de segurança, gravidade ou terapia. (19,6 Pontos)	549 (15,5)
**2. Investigação laboratorial: Bioquímica e microbiológica. (0 e 4,3 Pontos)**	
Presente	3530 (99,5)
Ausente	17 (0,5)
**3. Medicações, exceto drogas vasoativas. (0 e 5,6 Pontos)**	
Presente	3534 (99,6)
Ausente	13 (0,4)
**4. Procedimentos de higiene**	
4 a. Normal (4,1 Pontos)	1680 (47,4)
4 b. Realização de procedimentos de higiene que durem mais do que 2 horas em algum plantão. (16,5 Pontos)	1728 (48,7)
4 c. Realização de procedimentos de higiene que durem mais do que 4 horas em algum plantão. (20 Pontos)	139 (3,9)
**5. Cuidados com drenos. Todos, exceto sonda gástrica. (0 e 1,8 Pontos)**	
Presente	402 (11,3)
Ausente	3145 (88,7)
**6. Mobilização e posicionamento**	
6 a. Realização do(s) procedimento(s) até três vezes em 24 horas. (5,5 Pontos)	362 (10,2)
6 b. Realização do(s) procedimento(s) mais do que três vezes em 24 horas ou com 2 enfermeiros em qualquer frequência. (12,4 Pontos)	2877 (81,1)
6 c. Realização do(s) procedimento(s) com 3 ou mais enfermeiros em qualquer frequência. (17,0 Pontos)	308 (8,7)
**7. Suporte e cuidados aos familiares e pacientes**	
7 a. Suporte e cuidado aos familiares e pacientes que requerem dedicação exclusiva por cerca de 1 hora em algum plantão tais como: explicar condições clínicas, lidar com circunstâncias familiares difíceis. (4.0 Pontos)	3458 (97,5)
7 b. Suporte e cuidados aos familiares e pacientes que requerem dedicação exclusiva por 3 horas ou mais em algum plantão tais como: morte, circunstâncias especiais (p. ex. grande número de familiares, problemas de linguagem e famílias hostis). (32,0 Pontos).	89 (2,5)
**8. Tarefas administrativas e gerenciais**	
8 a. Realização de tarefas de rotina tais como: procedimentos de dados clínicos, solicitação de exames e troca de informações profissionais (por ex. passagem de plantão e visitas clínicas). (4.2 Pontos)	2742 (77,3)
8 b. Realização de tarefas administrativas e gerenciais que requerem dedicação integral por cerca de 2 horas em algum plantão tais como: atividades de pesquisa, aplicação de protocolos, procedimentos de admissão e alta. (23,2 Pontos)	799 (22,5)
8 c. Realização de tarefas administrativas e gerenciais que requerem dedicação integral por cerca de 4 ou mais de tempo em algum plantão, tais como: morte e procedimentos de doação de órgãos, coordenado com outras disciplinas. (30,0 Pontos)	6 (0,2)
**9. Suporte respiratório. (0 e 1,4 Pontos)**	
Presente	3213 (90,6)
Ausente	334 (9,4)
**10. Cuidados com vias aéreas artificiais. (0 e 1,8 Pontos)**	
Presente	2083 (58,7)
Ausente	1464 (41,3)
**11. Tratamento para melhora da função pulmonar. (0 e 4,4 Pontos)**	
Presente	3291 (92,8)
Ausente	256 (7,2)
**12. Drogas vasoativas. (0 e 1,2 pontos)**	
Presente	1491 (42)
Ausente	2056 (58)
**13. Reposição intravenosa de grandes perdas de fluídos. (0 e 2,5 Pontos)**	
Presente	1242 (35)
Ausente	2305 (65)
**14. Monitorização de átrio esquerdo. Cateter de artéria pulmonar com ou sem medidas de débito cardíaco. (0 e 1,7 Pontos)**	
Presente	18 (0,5)
Ausente	3529 (99,5)
**15. Reanimação cardiorrespiratória nas últimas 24h. Exclui-se soco precordial. (0 e 1,7 Pontos)**	
Presente	11 (0,3)
Ausente	3536 (99,7)
**16. Técnicas de hemofiltração. Técnicas dialíticas (0 e 7,7 Pontos)**	
Presente	726 (20,5)
Ausente	2821 (79,5)
**17. Medida quantitativa do débito urinário. (0 e 7,0 Pontos)**	
Presente	3194 (90)
Ausente	353 (10)
**18. Medidas de pressão intracraniana. (0 e 1,6 Pontos)**	
Presente	18 (0,5)
Ausente	3529 (99,5)
**19. Tratamento de acidose/ alcalose metabólica. (0 e 1,3 Pontos)**	
Presente	988 (27,9)
Ausente	2559 (72,1)
**20. Nutrição Parenteral Total. (0 e 2,8 Pontos)**	
Presente	89 (2,5)
Ausente	3458 (97,5)
**21. Alimentação enteral por sonda gástrica ou outra via gastrointestinal. (0 e 1,3 Pontos)**	
Presente	2145 (60,5)
Ausente	1402 (39,5)
**22. Intervenção(**ões**) específica(s) na Unidade de Terapia Intensiva.**	
Presente	3213 (90,6)
Ausente	334 (9,4)
**23. Intervenções específicas fora da Unidade de terapia Intensiva. (0 e 1,9 Pontos)**	
Presente	514 (14,5)
Ausente	3033 (85,5)

A média de pacientes/enfermeiros foi de 3,27 (DP:±2,19), a média de pacientes /profissionais foi de 1 (DP:±0,62,), enquanto a média do NAS/enfermeiros foi de 14,63 (DP:±5,12) e a média do NAS/profissionais foi de 4,51(DP:±0,6).

Não houve associação significante dos desfechos alta e óbito com as relações: média de NAS por enfermeiros (p = 0,6233), média de pacientes por média de enfermeiros (p = 0,4884) e média de pacientes pelo número de profissionais (p = 0,6377).

Não houve associação do NAS com SOFA, com a idade, com o tempo de VM e tempo de UTI (p > 0,05). Entretanto, maiores escores do SOFA foi associado a maior idade dos pacientes, maior tempo de VM e maior tempo de internação na UTI (Tabela 3).

**Tabela 3:** Associação entre Nursing Activities Score e Sequential Organ Failure Assessment com idade, tempo de ventilação mecânica e tempo de internação na Unidade de Terapia Intensiva – São Paulo, Brasil, 2021 (n = 217).

		SOFA	Idade	Tempo de ventilação mecânica	Tempo de internação na UTI**
**NAS**	p-valor	0,6415	0,4524	0,7067	0,9784*
**SOFA**	p-valor	-	0,0007	0,0001	0,0001

*Coeficiente de Correlação de Spearman.

**Unidade de Terapia Intensiva.

Não houve associação entre a variáveis sexo (p = 0,1129), cor (p = 0,2126), IMC, (p = 0,1890), sedentarismo (p = 0,8531), tabagismo (p = 0,1686) número de comorbidades (p = 0,1015) e desfecho (p = 0,5743) com o NAS.

A associação entre SOFA e as variáveis sociodemográficas e clínicas demonstrou que os pacientes do sexo masculino tiverem escores maiores do que o sexo feminino, pacientes sedentários apresentam maiores escores no SOFA do que os não sedentários e os pacientes que foram a óbito obtiveram maiores escores do que os que receberam alta (Tabela 4).

**Tabela 4:** Associação entre Índice de gravidade Sequential Organ Failure Assessment e as variáveis sociodemográficas e clínicas, São Paulo, Brasil, 2021 (n = 217).

Variáveis	Sequential Organ Failure Assessment				p-valor
	N	Média (DP)	Mediana	Mínimo-Máximo	
**Sexo**					
Masculino	124	3,15 (2,37)	3	0-17	0,0141*
Feminino	93	2,46 (1,99)	2	0-11	
Í**ndice de Massa Corpórea**					
Magreza/Normal	47	3,3 (3,3)	3	0-17	0,9562**
Sobrepeso	77	2,74 (1,86)	3	0-9	
Obesidade/Obesidade grave	93	2,72 (1,81)	3	0-10	
**Cor**					
Branco	171	2,96 (2,29)	3	0-17	0,1384*
Não branco	46	2,43 (2)	2	0-9	
**Tabagista**					
Sim	8	3,13 (1,55)	3,5	0-5	0,3324*
Não	209	2,84 (2,26)	3	0-17	
**Sedentarismo**					
Sim	180	3,02 (2,05)	3	0-13	0,0002*
Não	37	2,03 (2,86)	2	0-17	
**Comorbidades**					
Nenhuma	41	3,02 (2,91)	2	0-17	0,0529**
1 a 2	101	2,45 (1,69)	2	0-9	
3 a 4	54	3,22 (2,15)	3	0-11	
5 ou mais	21	3,52 (2,94)	4	0-13	
**Desfecho**					
Alta	139	2,32 (2,03)	2	0-17	<0,0001*
Óbito	78	3,81 (2,27)	3	0-13	

*Teste de Mann-Whitney.

**Teste de Kruskal-Wallis.

O tempo de ventilação mecânica não se associou a idade (p = 1877), IMC (p = 0,3027), tabagismo (p = 0,6237) ou ao número de comorbidades (p = 0,2737). O tempo de internação na UTI se associou apenas com a idade, quanto maior a idade do paciente maior foi o tempo de internação (p < 0,0170).

Os desfechos óbito e alta se associaram à idade e número de comorbidades, onde pacientes que foram a óbito tinham uma média de idade maior do que os que receberam alta e os pacientes com cinco comorbidades ou mais apresentaram uma maior proporção de óbitos do que aqueles com menor número de comorbidades (Tabela 5).

**Tabela 5:** Associação dos desfechos alta e óbito com idade e variáveis clínicas – São Paulo, Brasil, 2021 (n = 217).

	Desfechos		p-valor
	Alta n (%)	Óbito n (%)	
**Idade**			
Média (±DP)	58,27 (±17,17)	69,77 (±14,61)	<0,0001*
Mediana	60	73,5	
Mínimo-Máximo	23-92	24-97	
**Índice de Massa Corpórea**			
Magreza/Normal	32 (68,1)	15 (31,9)	0,7246**
Sobrepeso	47 (61)	30 (39)	
Obesidade/Obesidade grave	60 (64,5)	33 (35,5)	
**Tabagista**			
Sim	4 (50)	4 (50)	0,4622***
Não	135 (64,6)	74 (35,4)	
**Comorbidades**			
Nenhuma	32 (78)	9 (22)	0,0421**
1 a 2	66 (65,3)	35 (34,7)	
3 a 4	32 (59,3)	22 (40,7)	
5 ou mais	9 (42,9)	12 (57,1)	

*Teste de mann-Whitney.

**Teste Qui-Quadrado.

***Teste exato de Fisher.

## DISCUSSÃO

Estudos realizados em pacientes com COVID-19 que necessitaram de internação na UTI apresentaram características sociodemográficas semelhantes aos achados deste estudo, onde a maioria eram homens, idosos, obesos e que possuíam comorbidades, apresentando ainda tempos semelhantes de VM e internação na UTI^(1,4,5)^. Estudo realizado a partir de dados de internação proveniente do MS (Ministério da Saúde), óbitos fornecidos pelo Registro Civil e dados populacionais do IBGE (Instituto Brasileiro de Geografia e Estatística) com o objetivo de identificar diferenças de mortalidade e hospitalização por sexo e idade em pacientes com COVID-19 demonstrou que os homens possuíam uma maior predisposição a não seguir os protocolos de isolamento e, desta forma, aumentavam a chance de contaminação e que os idosos devido à senescência e a presença de doenças crônicas, comuns nesta idade, aumentavam a chances de internação devido ao agravamento da doença^(16)^.

A medição da carga de trabalho da equipe de enfermagem, por meio do NAS, é muito importante para a adequação do número de profissionais por paciente, o que está associada a menores números de eventos adversos, menor número de infecções relacionadas à assistência à saúde e mortalidade hospitalar. Neste contexto, relaciona-se diretamente com a qualidade assistencial, garantindo segurança aos pacientes e a equipe assistencial^(8)^.

O NAS médio da população desse estudo foi de 84,79 o que equivale a 20,34 horas de assistência da equipe de enfermagem. Pesquisas realizadas com objetivo de demonstrar o impacto da pandemia na carga de trabalho da enfermagem, na Bélgica, na Itália e na Holanda, encontrou médias do NAS de 92, 84 e 55 respectivamente^(17)^. No Brasil, estudo realizado com pacientes oncológicos com COVID-19 obteve média do NAS de 110^(9)^ e outro com pacientes internados na UTI com COVID-19 obteve uma média de 86^(18)^. Neste contexto, estudos evidenciaram que pacientes com COVID-19 apresentaram médias maiores quando comparados aos pacientes sem o diagnóstico da doença. A análise dos itens assinalados do NAS neste estudo, nos permitiu concluir que a maioria dos pacientes necessitaram de mais horas de monitorização que o habitual, a utilização de maior número de profissionais para a realização de procedimentos de higiene e que a maioria deles precisaram de intervenções dentro da UTI, somados a frequente necessidade de VM, terapia de substituição renal e Oxigenação Extracorpórea (ECMO), o que pode justificar o aumento do NAS nesta população.

Em relação às principais atividades avaliadas por meio do NAS, os resultados são semelhantes aos encontrados em outros dois estudos^(8,19)^. A pesquisa desenvolvida na Bélgica com a participação de 95 pacientes e outra realizada nos Países Baixos com 218 pacientes, ambos evidenciaram que os pacientes com COVID-19 necessitaram de mais horas de monitorização, devido à gravidade dos pacientes e que este tempo pode ter sido acrescido também, pela necessidade da vestimenta complexa dos equipamentos de proteção individual. Demostraram também, que foi necessário um maior número de profissionais para procedimentos de higiene e mudança de decúbito, principalmente devido à posição prona, decúbito utilizado como padrão ouro para o tratamento dos pacientes com insuficiência respiratória grave. Outro aspecto semelhante, foi constituído pelos cuidados destinados aos familiares, devido à alta mortalidade e as limitações de visita durante a pandemia, aspectos que explicitam a complexidade do cuidado ao paciente com COVID-19.

Não foi encontrado estudo com a participação de pacientes com COVID-19 que realizaram a associação entre o número de enfermeiros/número de pacientes, número de profissionais/número de pacientes e o NAS/número de enfermeiros, entretanto estudo neerlandês com a inclusão de dados sobre a carga de trabalho de 29.445 pacientes coletados em UTIs de janeiro de 2016 a janeiro de 2018 demonstrou a associação entre a carga de trabalho com a mortalidade hospitalar^(20)^. Neste estudo o dimensionamento da equipe de enfermagem estava de acordo com o proposto pelo MS, COFEN e o proposto pelo NAS, ou seja, havia um cenário modelo para a prática assistencial segura e com qualidade^(10–12)^. Nesse contexto de dimensionamento de pessoal não houve associação desta relação com o tempo de VM, tempo de internação na UTI e mortalidade.

Neste estudo a carga de trabalho de enfermagem não foi correlacionada a gravidade dos sujeitos. No entanto, resultado divergente a este foi encontrado em pesquisa que evidenciou relação positiva moderada entre o NAS e o SOFA^(21)^. A divergência dos resultados pode ser explicada pela avaliação do SOFA apenas na admissão, diferente do NAS que foi realizada diariamente, talvez a coleta mais frequente do índice de gravidade seja necessária para melhor correlação entre essas variáveis.

Escores maiores do SOFA foram encontrados em pacientes com maior idade, os quais permaneceram maior tempo em VM e na UTI. Estudo realizado nos Estados Unidos, com a participação de cinco UTIs e a inclusão de 2320 pacientes com diagnostico de COVID-19, também encontrou a associação entre o SOFA e a idade^(22)^. Estudos têm demostrado a relação da idade com maior gravidade dos pacientes, ou seja, que os mesmos necessitarão de maior tempo de ventilação e maior período de internação^(1–5)^.

Revisão sistemática realizada com o objetivo de identificar fatores prognósticos de severidade e mortalidade em pacientes com COVID-19 com a inclusão de 207 estudos, demostrou que o sexo masculino foi um fator preditor de gravidade^(23)^. Estudos apontam questões sociais e biológicas como prováveis fatores determinantes para isto, visto que, há uma maior proporção de homens tabagistas, maior predisposição do sexo masculino a negligenciar a saúde, além disto, há diferença da resposta imunológica e celular entre os sexos, o que pode, desta forma, justificar maiores escores do SOFA entre os homens^(16,23)^.

Neste estudo o sedentarismo foi associado a maior gravidade dos sujeitos, resultado congruente com os de uma coorte realizada no Reino Unido com a inclusão de 387,109 adultos na qual foi demostrado relação entre prática de atividade física e gravidade por COVID-19. Indivíduos com pior estilo de vida apresentaram risco quatro vezes maior de agravamento da doença, pois há evidências de que a prática de atividade física possui efeitos benéficos no sistema imunológico, como os efeitos anti-inflamatórios e melhor resposta imune adaptativa^(24)^.

Neste estudo, maiores escores do SOFA foi encontrado entre os pacientes que foram a óbito, maiores escores do SOFA indicam disfunções severas ou de múltiplos órgãos, condições estas, encontradas comumente nos pacientes que desenvolveram a forma grave da doença. Apesar de não ter sido proposto para prever a mortalidade, estudos demonstram que um aumento no escore durante as primeiras 96h de UTI ou durante a internação associaram-se com maior risco de óbito^(14)^. Estudo realizado nos Estados Unidos com 320 pacientes com COVID-19 que desenvolveram sintomas respiratórios graves, evidenciou correlação entre o escore e a mortalidade, onde escore entre 0 e 1 foi associado a 100% de chances de sobrevivência e escores maiores que 11 a mortalidade em 100% dos casos^(25)^. Neste contexto, esse escore pode ser utilizado como parâmetro prognóstico dos pacientes acometidos pela COVID-19.

A associação entre tempo de internação e idade foi demonstrado por diversos estudos realizados com pacientes acometidos pela COVID-19, onde aqueles com idade mais avançada permaneceram por mais tempo internados^(1–5)^. O aumento de internação de idosos na UTI é um fenômeno esperado devido o envelhecimento populacional e a presença de doenças crônicas degenerativas, comumente encontrado com o avançar da idade. Idosos tem maior debilidade, menor reserva fisiológica, e o declínio e desregulação da função imunológica, conhecida como imunossenescência, resultam a uma resposta menos eficiente a infecção^(26–28)^. Desta forma, aqueles com maior idade, apresentam resposta imunológica menos eficiente, podendo demandar maiores cuidados e recursos, resultando em um tempo de recuperação prolongado, fazendo com que um maior tempo de internação nas unidades seja necessário, justificando esta associação.

Uma maior proporção de óbito foi encontrado entre aqueles com maior idade e que apresentavam um maior número de comorbidades. Dados mundiais demonstram mais de 3.4 milhões de mortes pelo COVID-19 até março de 2023, 2.4 milhões foram de pessoa com mais de 65 anos^(28)^. Coorte internacional, com a participação de 52 países e a inclusão de 600 mil pacientes demonstrou que acréscimo de 10 anos na idade do sujeito aumentou 1.5 vezes o risco de morte pela COVID-19^(28)^. O envelhecimento foi associado à elevação de biomarcadores específicos refletindo uma maior ativação endotelial, do sistema de coagulação, de citocinas inflamatórias e desta forma, dano aos órgãos^(29)^. Em relação as comorbidades, a presença de receptores do vírus em locais como pulmão, coração, sistema gastrointestinal e o aumento do número destes receptores em algumas doenças como asma e diabetes, eleva a vulnerabilidade de contaminação pelo vírus. Desta maneira, pode ocorrer a descompensação de doenças crônicas e resultar em disfunção de múltiplos órgãos e a morte destes sujeitos^(29,30)^. Assim, idade e comorbidades têm sido associado a desfechos desfavoráveis.

Avançamos desta forma, na avaliação do impacto da pandemia sobre a gravidade dos pacientes e consequentemente sobre a carga de trabalho de enfermagem, demonstrando as principais demandas destes pacientes em relação aos cuidados de enfermagem. Neste contexto, destacamos que o NAS foi uma ferramenta essencial para a gestão eficaz dos cuidados na UTI, assegurando assistência de qualidade para os pacientes gravemente enfermos, em um cenário de pandemia, que trouxe vários desafios para a equipe assistencial com as mudanças do perfil epidemiológico. Ainda, contribui para prevenir a sobrecarga dos profissionais, contribuindo para a saúde e o bem-estar dos profissionais de enfermagem, pois sua utilização promove equilíbrio entre demanda de trabalho e recursos disponíveis, favorecendo um sistema de saúde mais eficiente e seguro. A realização em centro único limitou o número de participantes, além disto, o fato de o SOFA ter sido avaliado somente na admissão do paciente pode ser uma limitação, quando associado a carga de trabalho, que foi avaliada diariamente.

## CONCLUSÃO

A carga de trabalho dos sujeitos desta pesquisa foi alta, exigindo 20,34 horas de assistência de enfermagem, a gravidade dos pacientes determinou o uso de mais horas de monitorização e maior número de profissionais para os procedimentos de higiene e mudança de decúbito. Não houve associação entre a carga de trabalho e os desfechos tempo de ventilação mecânica, tempo de internação na UTI, alta e óbito. Não foi encontrada associação entre o NAS e o SOFA, embora cálculos mais frequentes do índice de gravidade possam obter resultados diferentes. A gravidade do paciente foi associado as condições demográficas e clínicas, como a maior idade, maior número de comorbidades, maior tempo de ventilação mecânica e tempo de internação na UTI e uma maior mortalidade. A identificação da carga de trabalho da enfermagem tendo em vista o correto dimensionamento contribui para a qualidade assistencial e segurança para pacientes e profissionais.
